# Identification of Sleeper Support Conditions Using Mechanical Model Supported Data-Driven Approach

**DOI:** 10.3390/s21113609

**Published:** 2021-05-22

**Authors:** Mykola Sysyn, Michal Przybylowicz, Olga Nabochenko, Lei Kou

**Affiliations:** 1 Institute of Railway Systems and Public Transport, Technical University of Dresden, 01069 Dresden, Germany; michal.przybylowicz@mailbox.tu-dresden.de (M.P.); lei.kou@tu-dresden.de (L.K.); 2 Department of the Rolling Stock and Track, Lviv Branch of Dnipro National University of Railway Transport Named after Academician V. Lazaryan, 79052 Lviv, Ukraine; rsk_lf@diit.edu.ua

**Keywords:** ballasted track superstructure, sleeper support condition, dynamic simulation, track-side and on-board measurement, rail deflection, wavelet scattering, machine learning

## Abstract

The ballasted track superstructure is characterized by a relative quick deterioration of track geometry due to ballast settlements and the accumulation of sleeper voids. The track zones with the sleeper voids differ from the geometrical irregularities with increased dynamic loading, high vibration, and unfavorable ballast-bed and sleeper contact conditions. This causes the accelerated growth of the inhomogeneous settlements, resulting in maintenance-expensive local instabilities that influence transportation reliability and availability. The recent identification and evaluation of the sleeper support conditions using track-side and on-board monitoring methods can help planning prevention activities to avoid or delay the development of local instabilities such as ballast breakdown, white spots, subgrade defects, etc. The paper presents theoretical and experimental studies that are directed at the development of the methods for sleeper support identification. The distinctive features of the dynamic behavior in the void zone compared to the equivalent geometrical irregularity are identified by numeric simulation using a three-beam dynamic model, taking into account superstructure and rolling stock dynamic interaction. The spectral features in time domain in scalograms and scattergrams are analyzed. Additionally, the theoretical research enabled to determine the similarities and differences of the dynamic interaction from the viewpoint of track-side and on-board measurements. The method of experimental investigation is presented by multipoint track-side measurements of rail-dynamic displacements using high-speed video records and digital imaging correlation (DIC) methods. The method is used to collect the statistical information from different-extent voided zones and the corresponding reference zones without voids. The applied machine learning methods enable the exact recent void identification using the wavelet scattering feature extraction from track-side measurements. A case study of the method application for an on-board measurement shows the moderate results of the recent void identification as well as the potential ways of its improvement.

## 1. Introduction

**Figure 1:**
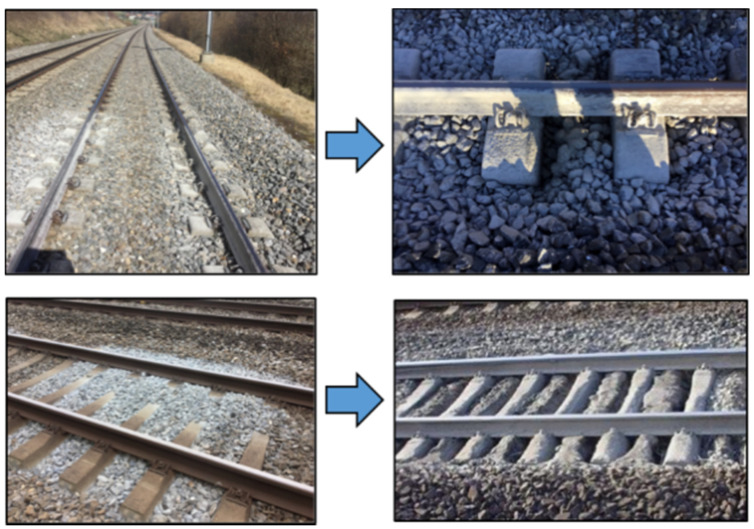
Poor sleeper support conditions and their consequences: ballast pulverization (**left**) and ‘mud pumping’ places (**right**).

The ballasted track superstructure is the most-used superstructure worldwide due to its low construction costs, flexibility of application, automated maintenance, etc. However, it has significant disadvantages due to the relatively quick deterioration of its geometric quality. The ballast layer has the highest influence on track geometry quality, which causes high maintenance costs. The costs consist of track inspection and corrective maintenance costs that, despite the high automation, still reach up to 30% of the overall maintenance costs [1,2]. The first stage of the ballast layer inhomogeneous settlements is related to the sleeper void initiation due to track local instabilities. The void accumulation causes unfavorable dynamic interaction in the contact zone between the sleeper and ballast bed [3,4], which results in accelerated void accumulation. Thus, the void zone can itself quickly be the source of local instabilities and track-bed damage such as ballast pulverization and separate track irregularities. The late removal of local instabilities can cause in the late stage significant and costly damages of the track subgrade such as ballast pockets and ‘mud pumping’ places (Figure 1). Therefore, the recent sleeper void identification using monitoring means is the best way to reduce the maintenance costs.

**Figure 2:**
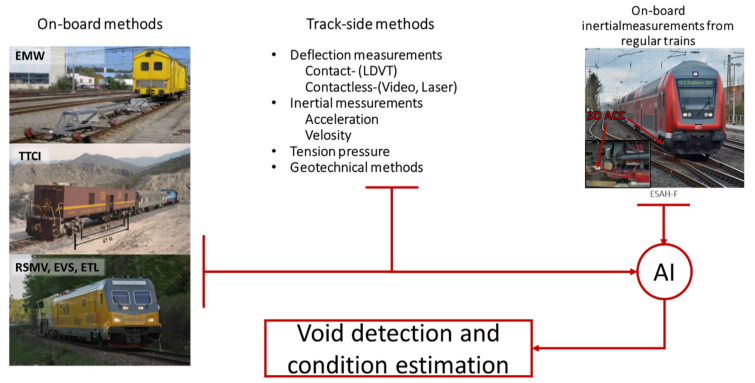
On-board [5,6,7] and track-side measurement methods for identification of sleeper support condition.

Nowadays, subgrade and sleeper support condition monitoring and estimation is presented by different on-board and track-side methods. The existing on-board monitoring methods (Figure 2, left) are usually referred to track stiffness measurement trains such as EMW-‘Einsenkungsmesswagen’ or the Swiss track stiffness measurement vehicle [5], RSMV or the rolling stiffness measurement vehicle of Swedish railway [6], TTCI or American track loading vehicle [7], CARS or Chinese track stiffness measurement system [6,7] and others. The on-board measurement of track stiffness is usually based on track deflection measurement under the different vertical loadings using special measurement trains. The methods provide an estimation of non-linear track-stiffness from which the void parameters can be detected and quantified. However, the on-board methods from measurement trains have many technical restrictions, such as the low train velocity and accuracy. Moreover, high measurement costs due to the necessity of the special measurement trains do not allow frequent enough measurements for tracking void development.

The present track-side methods for void identification on, more generally, stiffness measurement (Figure 2, center) is usually based on track deflection and inertial measurements under train or controlled loading. A method for track-side estimation of the sleeper support conditions is proposed in the study [8]. The method exploits the inertial measurements on one sleeper and the micro-tremor analysis to identity hanging sleepers. The detailed interpretation of the relation between the longitudinal level and sampled micro-tremor using data is demonstrated. A number of measurement techniques for evaluating track support are presented in [9]: track deflection techniques, video cameras, accelerometers, spectral analysis of surface waves, falling weight deflectometer and others. A comparison of the methods is demonstrated for improving inspection and maintenance at problem locations. Track stiffness identification in the transition zones using video recording, laser array, and geophone is presented in the work [10]. The method is based on the measurement of dynamic deflection under the rolling train on many sleepers along the track. The estimation of the subgrade linear stiffness was based on the beam on elastic foundation theory. An evaluation of the ballast support condition using the sleeper stress measurement is shown in the study [11]. The measurement of bending moments across the concrete sleeper were used for the back-calculation of the ballast support condition. To better quantify the variation of ballast pressure beneath the sleepers, the ballast pressure index is also proposed. A similar approach is proposed in the study [12], where a curvature-based damage detection method for the identification of the ballast voids under railway track sleepers is used. The curvature measurement is proposed using fiber-Bragg grating strain sensors. Similar applications of distributed fiber optic strain sensing for transport infrastructure are proposed in studies [13]. The paper [14] presented track-side monitoring of sleeper vertical deflection on a turnout. The monitoring is proposed to be stationary, and the void identification is based on artificial intelligence algorithms. Track support condition identification using inertial measurements is presented in the study [15]. The approach proposes the identification of sleeper deflection, track modulus and the at-rest position. The fast and robust identification of railway track stiffness from simple field measurement is proposed in the study [16]. The method is based on the measurement of the frequency response function under the excitations of hand-held hammers, which is relatively simple to implement and cost-effective. The data interpretation is based on the combination of a finite element track model and a data-driven Gaussian process regression model. The method enables fast predictions for large datasets and high accuracy of the identification. Another vibration-based method for ballasted track monitoring is presented in the study [17]. The information source was wireless inertial sensors embedded into the ballast particles. The structural health monitoring of the ballasted bed under an impact excitation was performed using spectral analysis. The presented track-side methods can provide accurate estimation of sleeper support condition. However, almost all track-side methods consider a portable application of the measurements systems that need costly manpower, whereas the application of the stationary track-side systems is connected to high hardware costs.

Another concept of track sleeper support monitoring is on-board measurement from regular trains (Figure 2, right). The concept is cost-efficient, different to the methods for the track-side monitoring of the track stiffness, as well as the measurement trains. It can be used for monitoring of the track network and can be frequent enough to track the void development. The most simple, reliable and cost-effective monitoring information is offered by using inertial measurements. An application of on-board inertial measurements from in-service trains for the identification of the sleeper support conditions is demonstrated in the studies [18,19]. The car-body vertical acceleration and GPS location measurements were analyzed by a multi-resolution analysis and state machine design methodology for the identification of the void zones. The multi-source data analysis ensured the rapid monitoring of the entire rail network. The proposed monitoring system improves safety and network performance by efficiently directing maintenance crews to the location of defects, minimizing time spent on maintenance and inspection. A similar measurement concept but with a more advanced analysis technique is proposed in the study [20]. The analysis exploits the sparsity inherent in train vibration data corresponding to excitation of the train’s fundamental mode. The sparse approach was validated on two monitored track sections over a 16-month period. The approach can only detect when track changes occur and offers insight into the type of such changes. An experimental case study [21] presents train bogie accelerations monitoring and their comparison to the maintenance measurements of track recording vehicles. The Hilbert transform is used to acquire the instantaneous amplitudes of the acceleration signals and signal energy level is proposed as a track condition indicator. The study indicates a significant influence of train speed on the condition indicator. It was shown that after taking into account the speed factor and corresponding scaling of the indicator, it matched the track recording measurements very well. A development of a method for estimation of the track quality using axle-box and car-body acceleration measurements is presented in papers [22,23]. A modified Karhunen–Loève transformation is used to extract the principal dynamics from measurement data. The investigation results showed that track condition estimation is possible with acceptable accuracy for in-service use and for defining cost-effective maintenance strategies. A cost-effective track condition monitoring system using embedded sensors on in-service vehicles is presented in the study [24]. The system is considered for quasi-continuous condition monitoring regarding short-wavelength—a few centimeters to a few meters—defects of railway tracks. The acceleration sensor data is combined with further relevant data such as the digital map of the railway infrastructure and other operational data. The relevant features from the axle-box acceleration data are extracted for pattern recognition and the further intelligent data analysis to provide spatiotemporal information about track conditions. Localization of the track irregularities using the axle-box accelerometer on in-service tram is presented in the studies [25,26]. The analysis was based on the extended Kalman filter application. Additional parameters such as vehicle velocity, GPS location and track defect images were monitored. An experimental investigation of the relationship between the irregularities recorded by a conventional track-geometry-measuring car and the resulting dynamic vehicle responses of axle-box accelerometer is presented in the paper [27]. It was concluded that the differences of the vertical axle-box accelerations and the lateral axle-box acceleration are practicably proportional. The study [28] describes the application of time-frequency analysis for condition monitoring of railway tracks from car-body acceleration measured in an in-service train. The Hilbert-Huang transform provided an indicator to diagnose track conditions from car-body vibrations as well as detecting track faults and identifying their type. The reviewed studies on track and sleeper support defect identification using inertial measurement from regular trains present different approaches for measurements and their interpretation. The measurements are presented by car-box-, bogie- and axle-box-mounted sensors that can provide different information quality with different measurement costs. Evidentially, the simple car-box-based measurements cannot replace more complicated axle-box ones but can be used for some long-wave track defects. There are also many interpretation methods presented. The most methods are based to spectral signal processing with different techniques to extract significant features that correspond to the defect type. The result of the interpretation is some dimensionless condition indicator for track state in some failure mode. Many studies evidence that the condition indicators correspond to the conventional indicators from track recording cars and therefore could replace them. However, the concept of inertial on-board monitoring has a fundamental disadvantage relating to the void measurement. It is not possible, as in the track-side methods, to measure rail deflections using only wheel trajectory measurements. Moreover, the measurements from regular trains are subjected to many uncontrolled factors that affect the data quality.

The present paper demonstrates an approach for the identification of the sleeper support condition. The aim of the study is the development of the methods that allow to differentiate between the void and geometrical irregularity based on track-side and on-board measurements. Different to other reviewed studies, the present research includes both mechanical modelling and a data-driven approach based on the developed method for efficient data acquisition from many void defect zones. The study is produced in the following steps:

- Mechanical modelling of track and vehicle interaction for cases of void and the equivalent geometrical irregularities;
- Exploration of differences in time and frequency domain between both cases of the irregularity;
- Wavelet scattering feature extraction and selection of the significant features from modelled recordings of axle-box acceleration;
- Development of the method for vertical rail deflection extraction from high-speed video recordings;
- Signal processing of the collected track-side measurements to extract the vibration features of wheel and rail oscillations in the void and reference zone;
- Statistical processing and void severity classification using machine learning methods;
- A case study of void identification on a common crossing from on-board measurements on regular trains.

## 2. Exploration of Void Influence of Track-Side and On-Board Measurements Using Mathematical Modelling

The objective of the section is the exploration of the dynamic behavior of the railway track with sleeper voids, its influence on track-side and on-board measurements using mathematical modelling and its principal differences to the behavior in a geometrical irregularity. There have been many papers published over the last decades devoted to modelling of track dynamic behavior in the void zone.

A theoretical study of the sleeper contact impact due to unsupported sleepers is presented in the study [29] using FEM modelling. A void depth up to a 1-mm gap for several hanging sleepers was simulated. The influence of one single hanging sleeper with a 1-mm void is estimated to cause the increase of the sleeper ballast contact force at the sleeper adjacent sleepers by up to 70%. Another three-dimensional FEM modelling of the effect of unsupported ties at transition zones is demonstrated in [30]. The substantial increase of tie-ballast impact forces by almost 200% is noted for tie-ballast gap heights of 2.0 mm. A detailed flexible track system model based on a multi-body system and finite elements techniques is presented in the study [31]. Hanging sleepers were considered in the calculation of the non-linear ballast sleeper interaction in the form of a bi-linear function. The impact of forces because of hanging sleepers on the vehicle and on the track were simulated. An analytical and finite-element solution to the problem of a vibrating beam, fully or partly supported by an elastic foundation is presented in the paper [32]. The low influence of the foundation stiffness and railpad stiffness on the sleeper bending-mode eigenfrequencies is stated. A multibody vehicle–track model assembly that couples the integration of the continuous and discrete system is presented in the research [33]. An uncontacted spring-damping element underneath the unsupported sleeper and the triangularly unsupported sleeper are taken into account by the model. The existence of a critical gap size causing the largest force is found and is estimated to be 2.5 mm for four unsupported sleepers. An iterative modelling of long-term differential settlements that considers sleeper void influence is shown in the study [34]. The track model is presented by a finite element Euler–Bernoulli beam coupled with a three-piece bogie. The track irregularity evolution in the zone of the dipped weld under the influence of rail pad stiffness and the void depth is studied using the model. A three-dimensional numerical model of track was used in the study [35] to explore the effects of unsupported sleepers on the dynamic behavior of railway track. The model has taken into account the prestressing forces, variations of the sleeper cross-section along its length, losing or recovering contact between the sleeper and the ballast layer. A model validation for the case of a void-free sleeper support is presented. A coupled 1D track model with MBS vehicle model is presented in the study [36]. The model is used for studying the effects of various train bogie patterns on the performance of ballasted railway tracks with unsupported sleepers as well as various relations of track-deformed state to sleeper support conditions. A 3D finite element method model application for studying the turnout dynamic phenomena of unsupported sleepers is demonstrated in paper [37]. Different support conditions, velocities and positions of unsupported bearers are simulated. The results show that the performance of fiber-reinforced foamed urethane bearers are promising. However, no experimental measurements were presented and compared to the simulation results. A three-dimensional coupled vehicle-ballasted track-subgrade interaction model is developed in [38], where the vehicle is modeled as a multi-rigid-body system and the track-subgrade interaction is modelled by the 1D finite element method. The model was used for simulation of the soil elasticity unevenness and the contact break due to hanging sleepers. An analytical frequency domain model is developed in [39] with a double periodicity layer, capable of dealing with both sleeper periodicity and arbitrary non-uniformity in track properties. The model is used to estimate the track geometry degradation due to a locally reduced support stiffness because of hanging sleeper along the track. The degradation is quantified using the parameter of the mechanical energy dissipated. The study has shown that the sleeper mass and railpad damping are irrelevant with respect to degradation. A computation scheme of the moving element method for the analysis of high-speed train-track dynamics is presented in the study [40]. This numerical approach is presented as more accurate and efficient as the standard FEM in dealing with various train-track dynamic problems. The research findings show that the highest maximum rail-wheel contact force is observed to occur when the two hanging sleepers are spaced apart by three to five fully supported sleepers. The approach of a distributed model of the support for 2D simulations based on the Timoshenko beam element over an elastic foundation is presented in the study [41]. The model, compared to the standard model with the four-degree Timoshenko element, is analytically appended with an additional degree of freedom of the vertical displacement. The improvement enhances the temporal response, avoids rail stiffening and also corrects the overestimation of the contact force as well as reducing the computational costs. The further improvement of the model is demonstrated in the study [42], where Timoshenko FE that includes internal degrees of freedom (iDoF) is presented. The use of iDoF efficiently corrects the non-physical response of rail accelerations and the proposed filtering criterion for accelerations removes the remaining non-physical response. The model can substantially improve the defect detection and health monitoring of railway vehicle and track components.

The presented literature review shows many studies on modelling the dynamic track behavior considering the sleeper support conditions. Most of the studies are based on more or less detailed FEM models with or without coupled MBS vehicle models. However, many studies over the last years show an increase of interest in robust models based on 2D beam models and even on analytical approaches. Such models can be better fitted to the description of complex short-time nonlinear and long-term interaction processes in track rather than the redundant geometrical dimension of the FEM models. Nevertheless, all models suffer from the lack of good experimental validation of the effects of the dynamic behavior in the voided zone.

**Figure 3:**
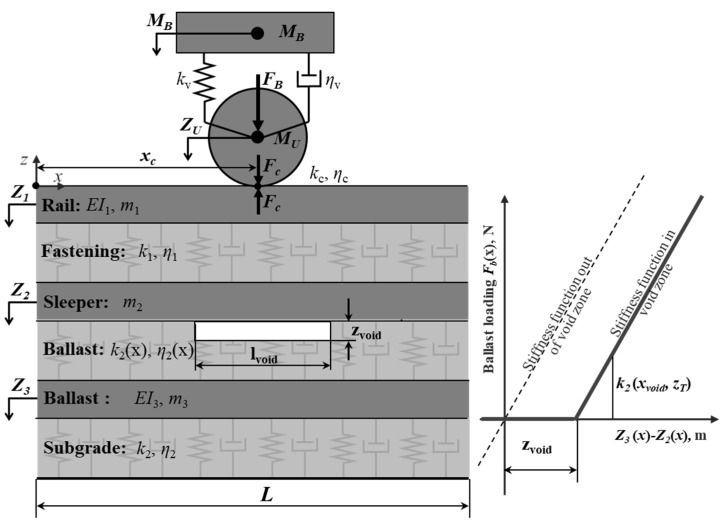
Three-beam model of track and rolling stock interaction.

The modeling in the present study is based on a three-beam model, whicht is coupled with a two-mass discrete model. The model is similar to that in the previous studies of the authors [43]. However, the discrete support of the separate sleepers was simplified to a continuous one in order to simplify the simulation result interpretation. A generalized mechanical system was used as a model (Figure 3, left). The system comprises two inertial bodies: sprung and unsprung masses corresponding to one axis and three continual inertial Euler–Bernoulli beams corresponding to track elements: a rail, a sleeper with fastening and a ballast layer with a ballast bed. The beam elements are separated by continual layers that correspond to the Winkler’s base and have viscous-dissipative properties.

The voids under the sleeper with length $l_{\mathrm{void}}$ and void depth $z_{\mathrm{void}}$ are taken into account as the gap between the second and third beam, whose foundation properties $k_{2}\left(x\right)$ and *η*_2_(*x*) depend on the void parameters. The interaction force between the beams is presented in Figure 3 (right) and described by the following relation:

(1) $$F_{b}=\{\begin{array}{c} -k_{2}\left(Z_{3}-Z_{2}\right)-\eta_{2}\left({\dot{Z}}_{3}-{\dot{Z}}_{2}\right),\;\;\mathrm{if}\;\left(Z_{3}-Z_{2}\right)>z_{\mathrm{void}}\;\\ 0,\;\;\;\;\;\;\;\;\;\;\;\;\;\;\;\;\;\;\;\;\;\;\;\;\;\;\;\;\;\;\;\;\;\;\;\;\;\;\;\;\;\;\;\;\;\;\;\;\;\;\;\;\;\;\mathrm{if}\;\left(Z_{3}-Z_{2}\right)\leq z_{\mathrm{void}} \end{array}\;\;$$

The numeric model of *n_x_* elements of a beam over the length *L* corresponds to the system of ordinary differential equations that in matrix form is written as follows:

(2) $$\left[M\right]\left\{\ddot{Z}\right\}+\left[C\right]\left\{\dot{Z}\right\}+\left[K\right]\left\{Z\right\}=\left\{P\right\}$$

where {P} is the vector of loadings; [*M*] is the matrix of element masses; [*C*] is the matrix of damping in connecting layers; [*K*] is the matrix of stiffness taking into account the bending rigidity of the beam; $\left\{\ddot{Z}\right\},\left\{\dot{Z}\right\},\;\left\{Z\right\}$ are vectors of accelerations, velocities and displacements of the beam elements, sprung and unsprung masses.

The finite difference method was used for the differential equation solution. The mass matrix [*M*] has a diagonal form with a non-zero main diagonal. It consists of two scalars *M*_U_ and *M*_B_ corresponding to unsprung and sprung masses and three square blocks [*M*_1_], [*M*_2_] and [*M*_3_] with dimensions *N* × *N* corresponding to the number of elements into which each beam is divided (3). The matrix of damping [*C*] (4) takes into account the interaction between the neighbor beams and the system of two vehicle masses. The contact interaction is introduced by the matrix $\left[\delta_{1j}\right]$, whose non-zero element of the main diagonal corresponds to the point of contact of the wheel and the rail.

(3) $$\left[M\right]=\left[\begin{array}{ccccc} M_{U} & 0 & 0 & 0 & 0\\ 0 & M_{B} & 0 & 0 & 0\\ 0 & 0 & \left[M_{1}\right] & 0 & 0\\ 0 & 0 & 0 & \left[M_{2}\right] & 0\\ 0 & 0 & 0 & 0 & \left[M_{3}\right] \end{array}\right],$$

(4) $$\left[C\right]=\left[\begin{array}{ccccc} \eta_{v} & -\eta_{v} & 0 & 0 & 0\\ -\eta_{v} & \eta_{v}+\eta_{\mathrm{Hz}} & -\eta_{c}\left[\delta_{1j}\right] & 0 & 0\\ 0 & -\eta_{c}\left[\delta_{1j}\right] & [\eta_{1}]+\eta_{c}\left[\delta_{1j}\right] & -[\eta_{1}] & 0\\ 0 & 0 & -[\eta_{1}] & [\eta_{2}]-[\eta_{1}] & -[\eta_{2}]\\ 0 & 0 & 0 & -[\eta_{2}] & [\eta_{3}]-[\eta_{2}] \end{array}\right]$$

The stiffness matrix [K] includes the additional diagonal elements near the main diagonal because of the bending rigidity and the combining layers (5). The matrix operator $\left[D^{4}\right]$ means decomposition of the bending stifness into a difference scheme.

(5) $$\left[K\right]=\left[\begin{array}{ccccc} k_{v} & -k_{v} & 0 & 0 & 0\\ -k_{v} & k_{v}+k_{\mathrm{Hz}} & -k_{c}\left[\delta_{1j}\right] & 0 & 0\\ 0 & -k_{c}\left[\delta_{1j}\right] & \left[k_{1}]+k_{c}\left[\delta_{1j}\right]+EI_{1}[D^{4}\right] & -[k_{1}] & 0\\ 0 & 0 & -[k_{1}] & [k_{2}]-[k_{1}]+EI_{2}\left[D^{4}\right] & -[k_{2}]\\ 0 & 0 & 0 & -[k_{2}] & [k_{3}]-[k_{2}]+EI_{3}\left[D^{4}\right] \end{array}\right]$$

The vector column of external loading {P} considers the function of forces from vertical geometrical irregularity and gravitational loadings. The initial and boundary conditions take into account the following physical content: the beams are fully restrained at the edges at any given moment and the element velocities are zero at the initial time. In order to avoid calculation error due to the boundary conditions, the wheel motion zone on the beam is located at a distance of more than 4 m outside from its ends. The model length is 25 m with the length discretization 1 sm. The overall number of degrees of freedom for three beams is 7500.

The model parameters presented in Table 1 are selected to provide the qualitative agreement with the experimental measurements [4], the reference information about the superstructure elements [2] and parameter estimation from other studies [31,34,43]. The properties of the model beams and the vehicle masses are considered for one rail and wheel. The superstructure parameters correspond to rail UIC60, half sleepers B70 and fastening pads Zw.700. The loading of the vehicle corresponds to the wagon with a train velocity of 120 km/h.

**Figure 4:**
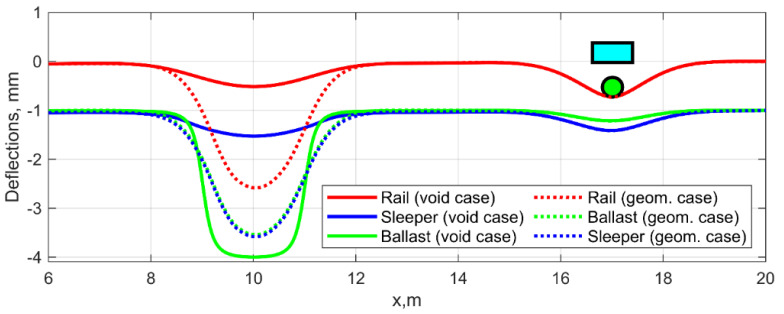
Elastic deformations of beam elements for the void and equivalent geometrical irregularity.

Experimental measurements [4] show that track dynamic behavior is characterized by complex dynamic interaction. The purpose of the modelling is to explore the features of the dynamic behavior of the railway track with sleeper voids and its difference to that caused by geometrical irregularity for the track-side and on-board measurements. Therefore, the track irregularities, which are applied in the model, are presented with void and geometrical irregularity. The void irregularities usually have the length $l_{\mathrm{void}}\;$up to 4 m and the depth $z_{\mathrm{void}}\;$up to 6 mm [4]. The depth and length values for the simulation case are $l_{\mathrm{void}}=3$ m and $z_{\mathrm{void}}=3\;$mm. The form of the void irregularity is unknown. Most of the studies assume the rectangular form of the void, i.e., the constant over the length and step-like change to the state without the void. However, the experimental measurements show that there are some gradients over one to two sleepers of the void depth at both sides of the void irregularity. Therefore, it is assumed in the model that the void irregularity form is chosen as flattened sinus function with the void variation over two sleepers at both sides. Figure 4 depicts the deflections of three beams and the void irregularity as the gap between the second (sleepers) and the third (ballast) beam. The lines of the beams are shifted along the vertical axis to provide a better illustration of the irregularities and deflection.

The simulated geometrical irregularity is chosen considering the research aim of the void identification from the other irregularities. Therefore, the case of the equivalent to void geometrical irregularity is determined using the quasistatic passing of the model for the void case. The equivalent to void geometrical irregularity corresponds to the wheel trajectory line and is shown in Figure 4, summed to the deflection of all three beams.

**Figure 5:**
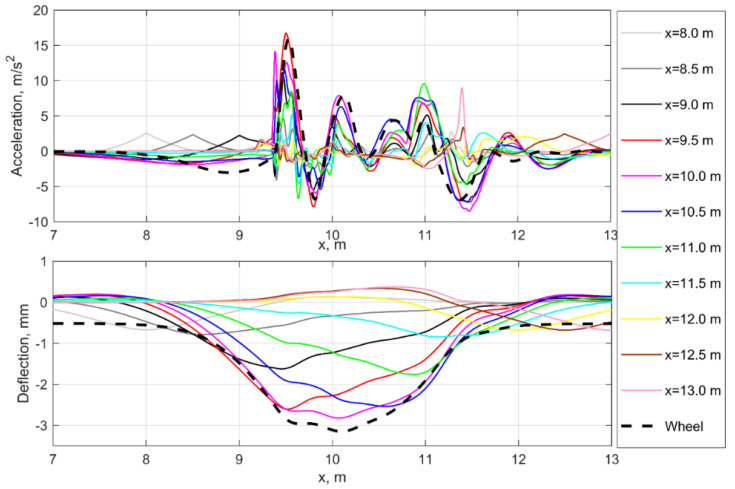
Simulated rail and wheel acceleration (**top**) and deflections (**bottom**) for the void irregularity.

The simulation results are presented as the track deflection and accelerations corresponding to on-board and track-side measurements. Figure 5, top shows the simulated measurements of rail deflections and accelerations in the different track sections relative to the voided zone as well as the wheel accelerations and trajectory in the track axis coordinate system. The rail deflections (Figure 5, bottom) show the features of the experimental measurements [4]: one or two kinks or disturbances on the wheel trajectory before the peak of maximal deflection. The deflection disturbances position corresponds to the acceleration maximal peaks position in Figure 5, bottom. Such behavior is explained by the dynamic impact of sleepers in the moment of void closure, which occurs during wheel movement over the voided zone beginning. Thereby, an additional second impact can appear after the first one as well as the disturbance on the unloading line, depending on the void parameters and vehicle velocities. While the deflection processes differ noticeably depending on the measurement section position along the track, the acceleration processes more similar along the track. Similar impact interaction in the void zone of a rail joint is confirmed experimentally by the field studies [44,45] where the sleeper ballast loading was directly measured by loading cells.

The wheel trajectory has a similar process as the rail deflection one for the track section in the middle side of the void. The similarities of wheel and rail dynamic behavior in the voided zone are clearly presented on the acceleration diagrams, explained by the mutual oscillation of wheel and rail while passing the void zone. Thus, the dynamic behavior due to the voided zone is present in both track-side and on-board measurements, which can be used for void monitoring and identification.

**Figure 6:**
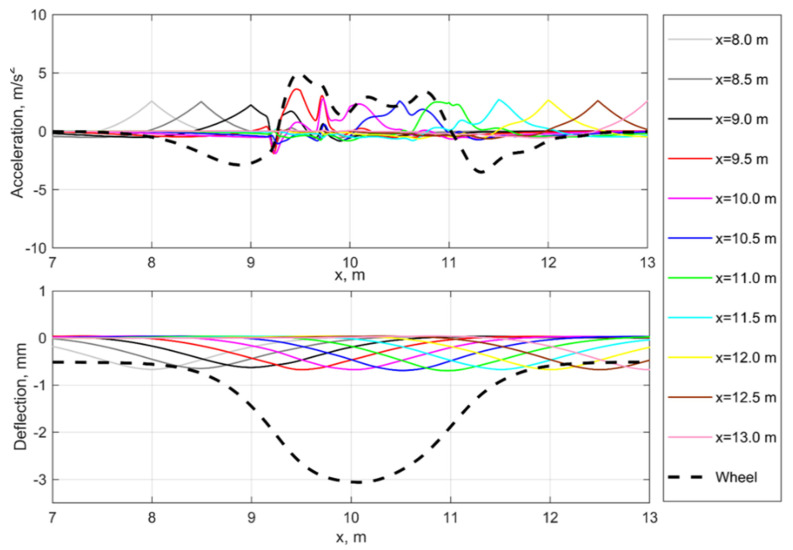
Simulated rail and wheel acceleration (**top**) and deflections (**bottom**) for the equivalent to void geometrical irregularity.

The simulation results for the equivalent to void geometric irregularity are presented in Figure 6. The main difference between dynamic interaction in the geometrical irregularity are the rail deflections (Figure 6, bottom) that are almost the same outside the irregularity while the wheel trajectory is similar to that of the void irregularity. The rail and wheel accelerations in Figure 6, top show more evident dynamic behavior; however, the maximal acceleration is more than three times lower than for the void irregularity. Additionally, there are no clear similarities between the wheel and rail acceleration processes. Thus, there are clear differences between the time-domain behavior in the void and the geometrical irregularity.

**Figure 7:**
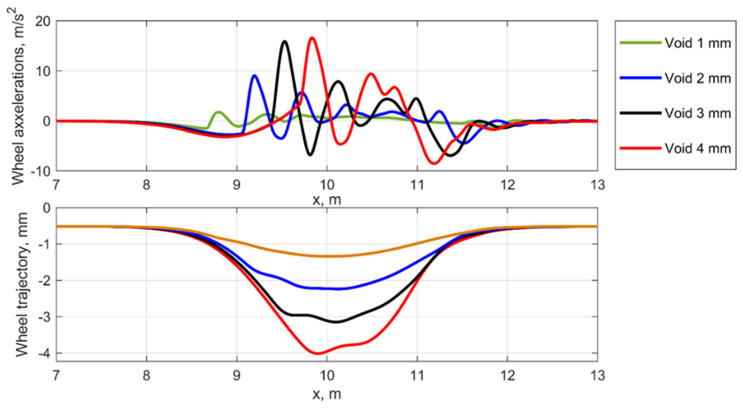
Simulated wheel accelerations and trajectories for the void irregularity.

**Figure 8:**
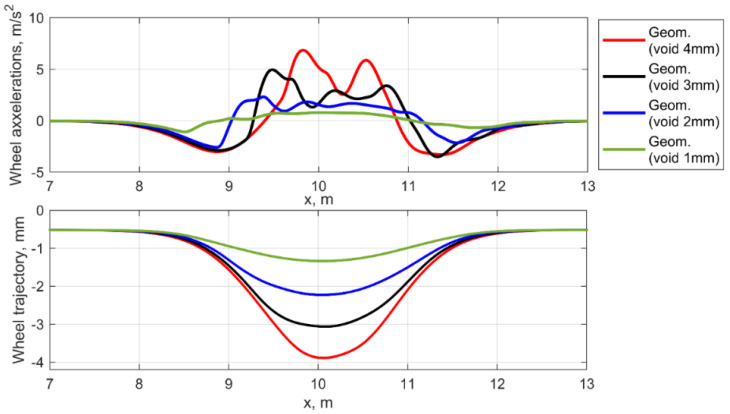
Simulated wheel accelerations in the geometric irregularity.

To provide a consistent analysis of the dynamic behavior, a comparison of the wheel trajectory and accelerations for different depths of void and the equivalent geometrical irregularities is presented in Figure 7 and Figure 8. The analysis shows the similar dynamic behavior in the voided zone Figure 7 for different void depths. The impact while entering the voided zone and the oscillations while leaving the zone are present for each void depth nut with different longitudinal positions.

The wheel dynamic behavior in the equivalent geometric irregularities for the different void depth is different to the void case. The wheel oscillations are formed by the wave of the irregularity and some similarity to the impact interaction is caused by the applied equivalent irregularity derived from the void irregularity.

The presented analysis of the dynamic behavior in the void zone shows the differences to the similar geometric irregularity both from the viewpoint of the track-side and on-board measurements. Different to the track-side measurements, the on-board measurements do not allow to determine the deflection of the rail elastic wave. However, the wheel trajectory variation due to the rail deflection within the void depth can be theoretically identified using the features of the dynamic interaction in the void zone. The similarities of the track-side and on-board measurements enable to use the track-side measurements for the identification of the voids from on-board ones after filtering the low frequency part of rail elastic wave deflection.

## 3. Spectral Analysis and Feature Extraction Using Wavelet Scattering

To explore the time-frequency differences of the simulated accelerations, wavelet scattering analysis is used. The research approaches in mechanical and railway engineering over the recent decades are characterized by a growing interest in applying wavelet frameworks to signal processing. The continuous wavelet transform method and global wavelet power spectra were used for evaluating degradation at railway crossings using axle-box acceleration measurements in [46]. A data-driven method that combines multiscale permutation entropy and linear local tangent space alignment is proposed in [47] to extract features of vibration signals to diagnose the faults of vehicle suspension systems. Suspension damage detection of the railway freight wagon by using CWT and spectral features is presented in experimental studies [48]. Study [49] proposed a feature extraction method to extract the root mean square distribution of decomposed ABA signals on a balanced binary tree as orthogonal energy features using the Hilbert transform and wavelet packet decomposition. An application of Hilbert-Huang transform is used to extract the time-frequency features of acceleration components of common crossing in an experimental investigation [50]. The wavelet packet entropy approach is proposed in the research [51] to detect local irregularities of railway catenary with different scales in length. A maximal overlap discrete wavelet packet transform and Shannon entropy estimates were used to extract the spectral features in the study of common crossing deterioration [52]. The paper [53] introduces the experimental investigation of common crossing deterioration using multifractal analysis to extract the features of Holder exponents and cumulants of the scaling exponents from track-side acceleration measurements. The application of the Wigner-Ville distribution based on feature extraction is presented in the studies of rail surface defect detection [54,55] and track irregularities [56]. Another application of Wigner-Ville transform for wheel-flat and rail surface defect identification is shown in [57]. The modal frequencies identification approaches are presented in the theoretical and experimental studies [58,59]. The approaches provide a solution for the identification of the operational modal frequency of track sleeper support based on the monitoring data.

The wavelet analysis has the benefit of multilevel and flexible time frequency resolution that results in the advantage of time localization and stability over Fourier transforms. However, it has more computational complexity and discrete wavelet transform or wavelet packet decomposition do not separate the carrier and modulated frequency on amplitude modulation [60]. The ensemble empirical mode decomposition with the Hilbert transform can separate the carrier and amplitude frequencies in amplitude modulation but it has high computational complexity. The Wigner-Ville distribution offers very high resolution in both time and frequency. However, for a mixture of several signal components in a signal, the Wigner-Ville distribution results can be difficult to interpret. This limits its application for many practical signals.

The wavelet scattering transform (WST), applied in the present study, forms translation and time-invariant, stable, and informative signal representations. It is stable to deformations and preserves class discriminability, which makes it particularly effective for classification. The WST has wavelet advantages with the application of a scaling window and a complex wavelet operator cascaded by averaging convolution to extract information. Moreover, it requires less computation, signal energy can be concentrated at a low bandwidth, features can be extracted at any layer.

**Figure 9:**
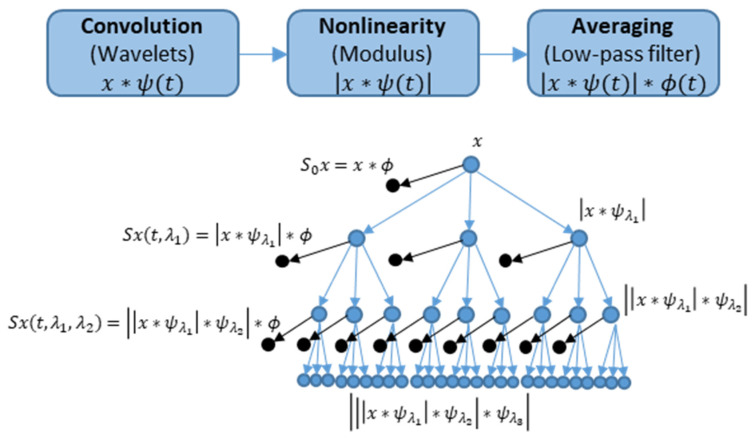
Wavelet scattering transform processes [60] (**top**) and wavelet scattering network [61] (**bottom**).

The overall process of WST consists of three main operations: convolution, nonlinearity and averaging, as described in Figure 9, top [61]. The input data are presented by signal x, a wavelet function ψ(t) and an averaging low-pass filter ϕ(t) or scaling function. The WST coefficients are determined by averaging of wavelet modulus coefficients using a low-pass filter ϕ(t). Figure 9, bottom presents the hierarchical representation of wavelet scattering coefficients at multiple layers [62,63]. A sequence of edges in Figure 9, bottom from the root to a node is referred to as a path. The tree nodes are the scalogram coefficients. The scattering coefficients are the scalogram coefficients convolved with the function ϕ(t). The set of scattering coefficients are the low-variance features derived from the data. The scattering transform generates features in an iterative way. At first, the data with the scaling function $x\cdot \phi_{\lambda 1}$ are convolved to obtain the zeroth-order scattering coefficients$\;S_{1}$. The following iteration corresponds to the (Figure 9, top): the wavelet transform of the input data with each wavelet filter in the first filter bank, the modulus of each of the filtered outputs, and averaging each of the moduli with the scaling filter. The results are the n-order scattering coefficients $S_{n}$.

**Figure 10:**
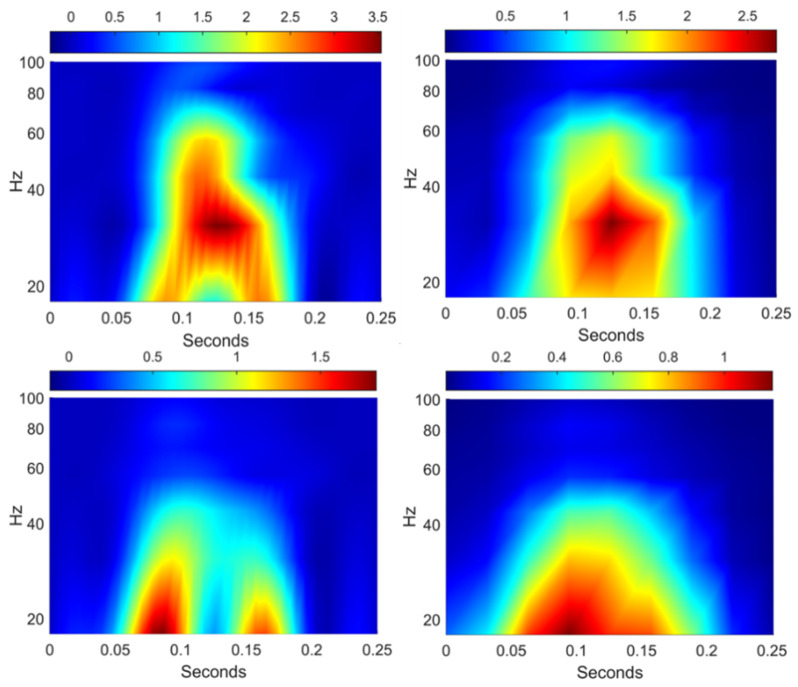
Wavelet scattering scattergrams (**right column**) and scalograms (**left column**) of the void 3 mm (**top row**) and the equivalent geometric (**bottom row**) irregularities.

The Matlab wavelet scattering tools [63] were used to analyze the acceleration of the acceleration signals for the case of the void irregularity and that of the equivalent geometrical one. The tool uses a wavelet time scattering decomposition with two filter banks. The first filter bank has a quality factor of eight wavelets per octave. The second filter bank has a quality factor of one wavelet per octave. Figure 10 presents the results of wavelet scattering transform for two cases of irregularities 3 mm in form of the time-frequency presentation of first-order scalogram and scattering coefficients. There is an obvious difference between the wavelet power spectrum distribution of void (Figure 10, top, left) and geometrical irregularities (Figure 10, top, left). The geometric irregularities are presented with two different zones with power spectrum maximal values with frequencies under 27 Hz, while the maximal values for void irregularity are located between 25 and 60 Hz in one zone corresponding to the impact zone. The diagrams of the scattering coefficients differ from that of the scalogram. The scalogram coefficients extract the high frequency or detailed components, while the scattering coefficients extract the low frequency components at every level of resolution [63]. Therefore, the variation of the scattering coefficients is lower than for the scalogram. However, the spectrum maximal values have the same time-frequency location both for the scalogram and the spectrogram presentation.

**Figure 11:**
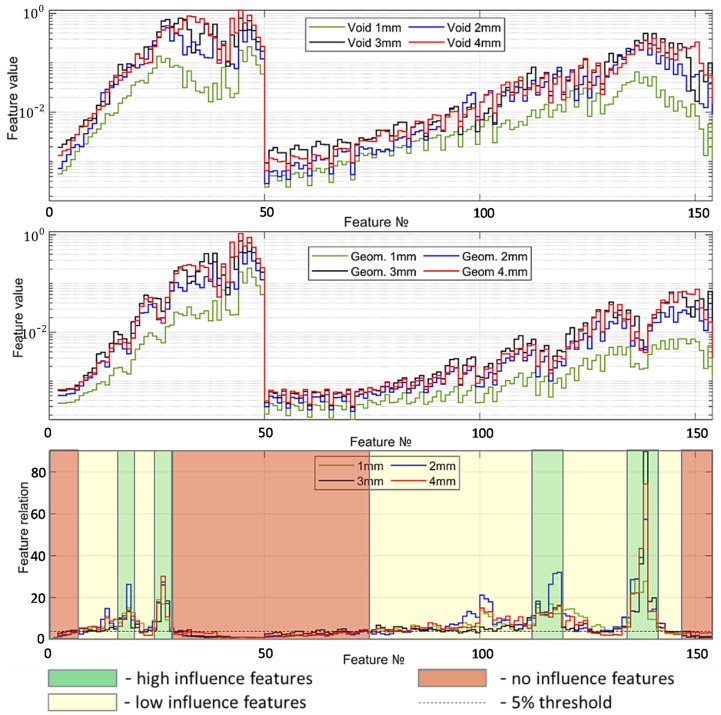
Wavelet scattering features for the void irregularities (**top**), the equivalent geometric ones (**center**) and the feature importance (**bottom**).

Before extracting the features from the modelled signals, the signals are oversampled to correspond to the experimentally determined signal length. The features of wavelet scattering coefficients are extracted for the number of resolutions across all orders of the scattering transform and the resolution of the scattering coefficients. The wavelet time scattering decomposition is constructed with the two filter banks: eight wavelets per octave in the first filter bank and one wavelet per octave in the second filter bank. The overall number of extracted features is 1232 for the signal length 2100, which corresponds to the measured signal sets. The diagrams of extracted features for the cases of void irregularities and the geometrical one as well as their significance are presented in Figure 11. The feature values for the case of void zone (Figure 11, top) with the different void depth have a similar process. However, the feature line of 1-mm void depth shows systematic lower coefficients than the 2-, 3- and 4-mm lines. The features of the geometrical irregularity present much higher variation, both along the number of the feature and across the lines of the irregularity depth.

The region of interest for the void detection are features 10 to 25 and 122 to 128, where the difference between the 2-, 3-, 4-mm lines are the lowest, which can be used as a common void indicator. Figure 11, bottom presents an estimation of the features corresponding to the highest difference between the void and geometrical irregularity zone as the relation between the features. The remarkable regions are for the feature numbers 1 to 10, 28 to 75 and 145 to 154, where the relation between them is less than 5% of the maximal variation, despite the maximal absolute values of the features. Thus, the features can be excluded from following statistical analysis. On the other side, there are the feature groups with maximal difference of void and geometrical zones for all considered depth cases from 1 to 4 mm: 16 to 20, 24 to 28, 112 to 119 and 134 to 140. The features can be used as void indicators. Indeed, the major number of features (65%) present high relative difference, but not for all depth cases and with high variation along the feature number. The features can be selected in the statistical processing using feature selection techniques.

## 4. In Situ Measurements of Rail Deflection in Void Zone

The aim of the section is the development of an efficient method for in situ measurements and the collection of the statistical information to use for the development of the method for void identification and classification using track-side and on-board measurements. The in situ information collection from many different void zones is based on high efficiency multipoint photogrammetric measurements with low-cost, high-speed cameras. Additionally, control measurements with conventional methods were applied for the photogrammetric method validation.

Image and video processing methods in the photogrammetric measurements are often used for railway and civil engineering applications. The application of a system of synchronized high-speed cameras to measure absolute longitudinal and vertical rail displacements using DIC is presented in [64]. The DIC system was used at track sites with a high-quality subgrade and one with soft subgrade, and for different train types. The data from one of the sites were used to determine parameters for system stiffness and damping. The deflection measurements had a good-quality geometrical quality despite the marker free measurements, but the time resolution of 100 frames per second could be too low to identify the impact interaction due to voids. Additionally, the method is complicated and time-intensive and costly for the frequent monitoring purposes. The monitoring of a railroad bridge under the vehicle load using DIC and accelerometers is presented in [65,66]. The accelerometer data is used to calculate displacements comparing to the DIC measurements to assess the accuracy of the accelerometer measurements. The research findings show that the used hardware together with DIC deflection estimation is an effective tool for low-frequency bridge displacement monitoring. A paper [67] presents a two-camera method for suppressing the camera vibration when measuring track displacements using DIC. The two-camera method was found successfully to reduce error due to camera movement while removing the subjectivity of choosing a cut-off frequency for filtering.

The application of the photogrammetric method for the diagnostic of transition zones is presented in [68,69]. The research [68] demonstrates the results of a field monitoring study of a railway bridge transition with void defects using the measurement both track displacements using digital image correlation and distributed rail strains using a Rayleigh-based fiber-optic analyzer. It was concluded that measurements of rail–sleeper gaps could be used to obtain a first-order estimate of the shape of the differential track settlement profile. However, the used high-speed cameras could not fully reflect the dynamic interaction in void zones. A similar photogrammetric approach is presented in the study [69], where the experimental analysis was performed in three transition zones in various conditions. By comparing the multiple-point displacements in the approaching zones in different conditions, it was found that the dynamic profile of the rail displacements has a good correlation with the track condition. An approach to use 3D DIC and pattern projection is proposed in [70] to assess the deformation of railway sleepers using on-board measurements. The approach is able to measure the crosstie’s full-field displacement and shape. A resolution of approximately five microns is estimated to measure the relative tie deflections, both inside and outside the rail area. The time resolution was 100 frames per second.

Another trend in the application of image and video-processing methods is related to deep-learning computer vision algorithms such as YOLO3 and others [71,72,73,74,75]. They provide the object recognition, that in case of the sleeper support diagnostics could automatically bring the necessary information about the loading position, loading type, image quality improvement, etc.

Most of the presented photogrammetric measurements studies are usually directed on a deflection measurement of a single point at one object with one camera. However, analysis of multiple points of relative motion could provide promising results, such as those presented in the studies [76,77]. The photogrammetric analysis of rail microhardness profile could deliver the information about the crack evolution. The spectral methods for surface analysis using fractional spline wavelets [78] could help to extract the significant features of the state. The photogrammetric measurement of the longitudinal rail curvature could potentially provide the distribution of loadings on the rail support and the sleeper stiffness.

**Figure 12:**
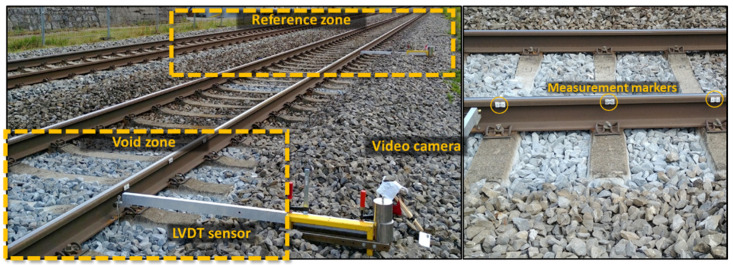
Experiment set-up for rail deflection measurement.

One of the most significant limitations of the reviewed studies is the low time-resolution of the used cameras that does not allow to acquire information about the dynamic impact of the sleepers on the ballast in the void zones. Additionally, most of the studies were used for a limited number of measurements due to the method complexity, which did not allow to collect the statistical information. The limitations are taken into account in the present study. The proposed method for the sleeper void identification is based on experimental information collected in track-side measurements of multipoint rail deflection. The high-speed video recording measurements were carried out in 15 different problem zones of Swiss railways (SBB) by infrastructure managers. The measurement evaluations were produced by ETH Zürich [79] and TU Dresden in the present work. The digital image correlation (DIC) method was used for extracting the rail deflection information. The results of the video measurement method were compared in three zones with the conventional measurement method by using the LVDT sensor that was placed on the console beam on the stable basis. The video recordings were carried out using two high-speed SONY DSC-RX0 cameras with an acquisition frequency of 1 kHz and an image resolution of 1920 × 1080 pixels. The recordings in each problem zone were realized using two high-speed cameras for the damaged zone and undamaged reference zone, 8 to 10 m away from the first one (Figure 12). The cameras were located on one side of the track, at 150 cm distance from the rail to have three markers in the field of view. This allowed a resolution of 0.61 mm/pixel.

**Figure 13:**
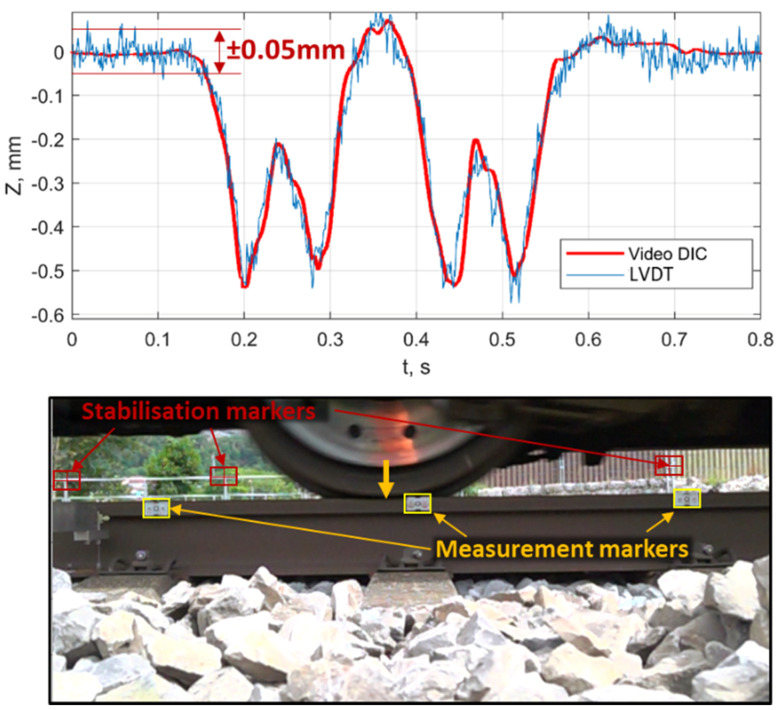
Rail deflection measurements using DIC method. (**top**) Comparing video DIC and LVDT methods. (**bottom**) Markers in the space between the wheelsets.

The measurement of rail deflection using high-speed video recordings is a cost-effective alternative to the LVDT (linear variable differential transformer) measurement, which enabled the collection of statistical information. The application of the DIC method increases the accuracy of marker tracking from 0.61 mm/pixels to about ±0.05 mm, which makes the accuracy of the video method sufficient for low rail-deflections in the reference zones (Figure 13, top). Additional advantages of the video measurements are multipoint tracking and the stabilization of train-induced camera vibration using the fixed background markers in the space between the wheelsets (Figure 13, bottom). Moreover, the simultaneous tracking of the wheel position offers additional advantages for void identification since the location of the rail deflection maxima does not correspond to the wheels position while running over the void zone. The software for rail DIC tracking and the automatic tracking of the background markers was developed.

**Figure 14:**
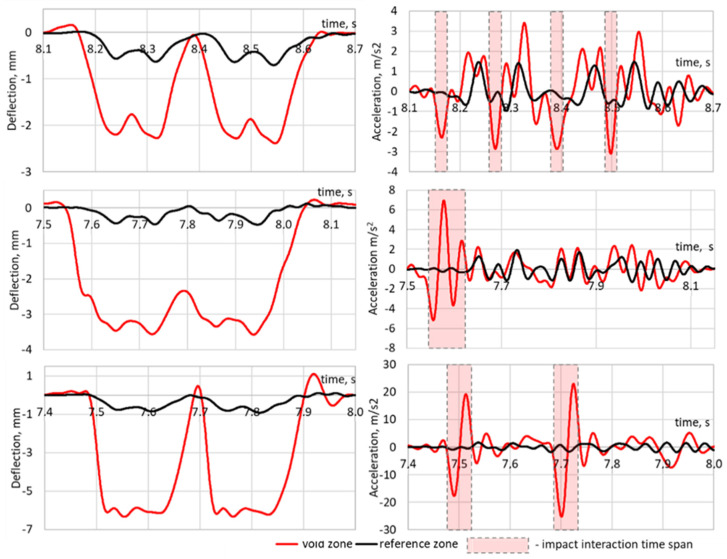
Measured rail deflections (**left column**) and the derived accelerations (**right column**) for different void damages: (**top**)—ICE1 110 km/h, (**middle**)—RBDe560 109 km/h, (**bottom**)—RABDe500—164 km/h.

The typical measurement cases for different void damage from low to high are shown in Figure 14. First of all, the voided zones are characterized by an increase in deflections. Additionally, an increased dynamic interaction is present due to the impact of the sleepers on the ballast bed. The impact usually has the highest value before the wheel maximal deflections or in the time while the wheel enters the void zone. It is especially peculiar for a void depth more than 3 mm. The rail accelerations for a 6-mm void depth can reach $\pm 25m/s^{2}$ or more than 10 higher times than for the reference track. The sleeper-ballast acceleration on the impact point could probably be higher than on the rail. The modelling results show that the sleeper acceleration during impact can reach 150 to 200$\;m/s^{2}$, which could accelerate the ballast settlements and void development.

## 5. Statistical Study of Relation between Sleeper Support Conditions

A data-driven approach is used to recover the relation of the measured rail accelerations and sleeper support conditions. The relation is used for the development of the method for sleeper void detection and quantification using track-side and on-board measurements.

Various data-driven approaches were proposed in numerous studies on railway infrastructure diagnostics over the last years. A one-dimensional convolutional neural network was proposed in the study [80] for damage detection of fastener clips using time-domain data recorded by accelerometers on the rail. The measurement data were acquired from laboratory impact tests for the track system under different health conditions of fastener clips. Additionally, numerical datasets were generated using vehicle track-coupled dynamics model in order to collect rail response induced by the passing train. A similar approach with 1D lightweight convolutional neural network architecture fitted by Bayesian optimization for wheel flat detection is presented in the study [81]. The acceleration measurements of a car-body acceleration using embedded systems was used for model training. The lightweight approach provides the best trade-off between accuracy and model complexity. An artificial neural network classifier has been used for automatic identification of the track circuits signal disturbances in the research [82]. We extracted the signal features before the model training using the wavelet packet energy Shannon entropy method. The proposed method shows high effectiveness of track circuit disturbance identification compared to the time-consuming and expensive visual analysis. The application of a long short-term memory (LSTM) network for switch gap size prediction is proposed in [83]. The regression and autoregressive integrated a moving average model to describe the change law of the switch gap size. The following LSTM network model predicts the switch gap temperature by referring the external weather forecast. The paper [84] presents a subgrade stability evaluation model based on a fuzzy neural network approach. The approach extracts correlations between input and output characteristic quantities that are used to construct the nonredundant mapping function between influencing factors of subgrade unstable deformation. An approach proposed in [85] uses a variational autoencoder (VAE) to detect the anomalies in the accelerations measurements from monitoring the longitude elevation of track geometry and the dynamic response of an in-service train. The approach has an advantage on distance- and density-based anomaly detection methods in robustness, preventing overfitting, and it does not require model-specific thresholds for detecting anomalies. A similar LSTM architecture is proposed in the study [86] to examine the measured time-series from on-board data of acceleration measurements. The proposed architecture investigated the characteristics of time-series signatures during a short period and classified the associated track segment to normal or defect states with an automated labelling method that used label defects for associated time-series signatures during the training phase. A convolutional neural network and extreme learning machine algorithm were used in studies [87,88] to detect different abnormalities in the axle-box measurements of the acceleration signal and their location using the global positioning system. The measurements were pre-processed before the model training by using a continuous wavelet transform. The paper [89] demonstrates three different approaches for combined defect detection and evaluation: deep neural network, convolutional neural network, and recurrent neural network. The simulated axle-box accelerations were used as statistical information for model training. The combined defects are presented with settlement and dipped joint geometrical irregularities. The DNN model used 14 extracted time-domain features as simplified data and CNN and RNN models were used without processing and data extraction. The three severity groups classification has shown up to 99% accuracy for the RNN model. However, the study was not supported with experimental measurements. Another deep learning-based approach for the detection of rail joints or defects by using convolutional neural networks is presented in [90]. The approach proposes a classification-based method to detect rail defects such as localized rail surface damage in joints, turning points, and crossings by using acceleration data from a rail inspection vehicle. Two convolutional networks—ResNet and fully convolutional networks—were investigated and evaluated with the collected acceleration data. The experimental results demonstrated that both approaches achieve good performance in joint detection. A nonlinear autoregressive neural network with principal component analysis information fusion was used in the study [91] to obtain the relationship between wheel tread wear and external related variables. The ways for further improvement of prognosis have been analyzed. A model for data-driven bias correction and defect diagnosis for in-service vehicle acceleration measurements is presented in the research [92]. The model consists of two parts: a bias correction sub-model, and a defect diagnosis sub-model for in-service vehicle acceleration measurements. The actual measurements data from the railway were used to validate the proposed model. The accuracy of vibration defect diagnosis was 75.8%. A statistical model based on the generalized extreme value distribution is proposed in the study [93] to develop the condition-monitoring system for the assessment of ballast quality in railway turnouts. The model describes the behavior of the resonance frequency associated with the ballast based on train-induced track vertical acceleration. The monitoring system consists of an EMD-based frequency estimator and a change detection algorithm based on a generalized likelihood ratio test.

The study [94] explores a variational heteroscedastic gaussian process approach while using variational Bayesian and Gaussian approximation for data modelling, estimation of the monitoring data uncertainty, and further data forecasting. The online SHM system using fiber Bragg grating (FBG) sensing technology was implemented on the basis of the approach. The approach provides more robust regression results and the estimated confidence level better depicts the heteroscedastic variances of the noise in measurements data. Another wayside fiber Bragg grating (FBG)-based wheel condition monitoring system for detecting the wheel tread defects is presented in the study [95]. The system is based on a Bayesian blind source separation method to extract the defect-sensitive feature from the monitoring data. The Bayesian approach is used in the study [96], which presents a data-driven prognostic methodology of common crossing residual life prediction. The methodology includes a machine learning approach, including feature extraction, selection, fusion and degradation modelling. The approach is based the statistical information of axle-box inertial measurements on operational trains from the whole lifecycle of one common crossing. A real-time defect detection methodology based on Bayesian dynamic linear model is shown in the paper [97]. The methodology consists of prognosis, potential outlier detection, change-point detection, and quantification of damage extent and uncertainty. The statistical information is acquired from 52 FBG strain sensors, four FBG temperature sensors, 11 tri-axial accelerometers and one noise sensor. The study [98] proposes a method for the automated detection and recognition of bridge defects using the singular value decomposition to formulate the feature vector set and the subsequent hybridization of the Elman neural network. The developed method outperformed other conventional prediction models. The model-based condition monitoring approach is proposed in the study [99]. The approach is realized in a tailored signal processing tool for operational condition monitoring.

Most of the presented studies are based on data-driven deep-learning approaches, spectral signal processing for feature extraction and either experimental or simulation-based sources of statistical information. However, only a few studies present both data-driven and model-based approaches together. The application of mathematical modelling and numerical simulation could potentially improve the data-driven approach. On the other side, some studies replace real measurements with the simulated ones without comparison to experimental measurements.

**Figure 15:**
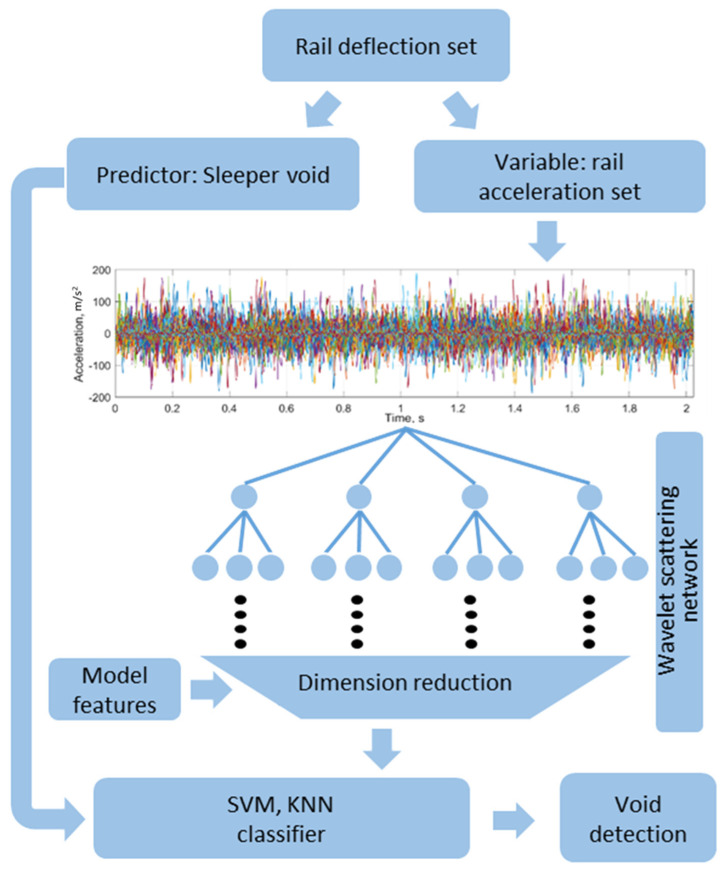
Flowchart of statistical processing for void identification.

The present statistical investigation is based on experimental data collected from 15 damage zones using the developed high-speed photogrammetrical tool for rail deflection measurements. Thereby, the simulation results of the track–train dynamic interaction in voided and reference zones as well as their time-frequency analysis are used to improve the data-driven approach. The improvement consists of the additional model-based feature selection. The flowchart of the statistical processing is presented in Figure 15. The rail deflection dataset of 652 time series is separated in two independent variables: the vector of maximal sleeper void and the corresponding rail acceleration series dataset. Thereby, the Winkler-line rail deflections process is removed from rail deflections to avoid the bias to predictor variable. An additional reason for the high-pass filtering is the intention to investigate the vibration information that is the same both for the track-side and on-board measurements. Moreover, the study in the section modelling has shown the similar dynamic vibration due to dynamic impact in the void zone for both measurements.

The acceleration time series dataset is in the second step processes to extract time-frequency domain features using the previously constructed wavelet scattering network. After the feature extraction, the dimension is reduced from 2000 time-series variables for the input data to 154 wavelet scattering features. After the following model-based feature selection, the number of features is reduced to 88. In the next step, two supervised machine learning approaches were used: support vector machine (SVM) and k-nearest neighbor classifier (KNN). The main reason why the machine learning methods were preferred to the popular deep learning ones is the relatively small dataset of the experimental measurements. Additionally, the machine learning models are simpler in interpretation.

**Figure 16:**
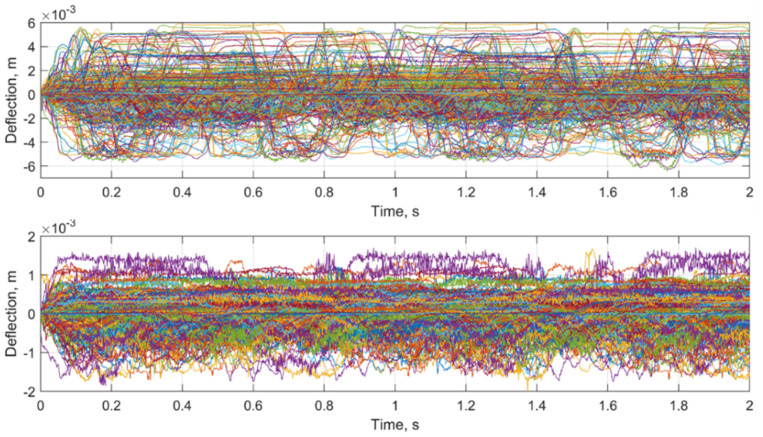
The raw deflection time-series of rail in the void (**top**) and reference zone (**bottom**).

The rail deflection dataset consists of two groups: the first for the measurements in the various ballast damage zones and the second for the simultaneous measurement in the reference zone, 10 outside the void zone. Each measurement zone is equipped by three markers for rail deflection measurements. The measurements were carried out in each measurement zone for about 12 train passages of different train types with velocities in range the of 100 to 160 km/h. The overall dataset of successful measurements is 652 time series. The comparison of the time-series datasets for the void and reference zone is presented in Figure 16. The presented rail deflection lines are relative to the initial measurement point. The measured deflection range in the void zone (Figure 16, top) is up to five times higher than in the reference zone (Figure 16, bottom).

**Figure 17:**
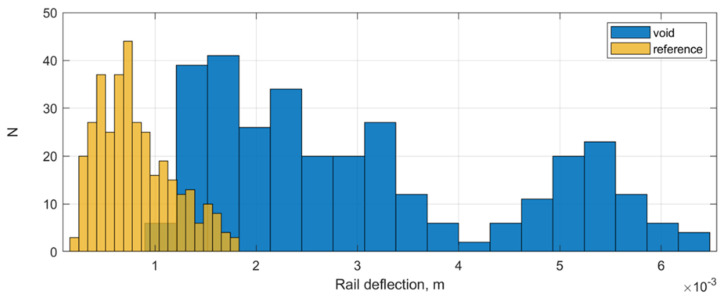
The distribution of rail deflection in the voided and reference zone.

The histograms of maximal rail deflection distribution in the voided and reference zone are depicted in Figure 17. The diagrams show that the ballast damage zones are characterized by a wide maximal deflection distribution from about 1.5 mm up to 6 mm. The reference zones have the most measured deflections in a range up to 1 mm but some deflections can reach up to 2 mm. The reason is the influence of the damage-independent factors on the rail deflection such as various subgrade stiffnesses and various superstructure types.

**Figure 18:**
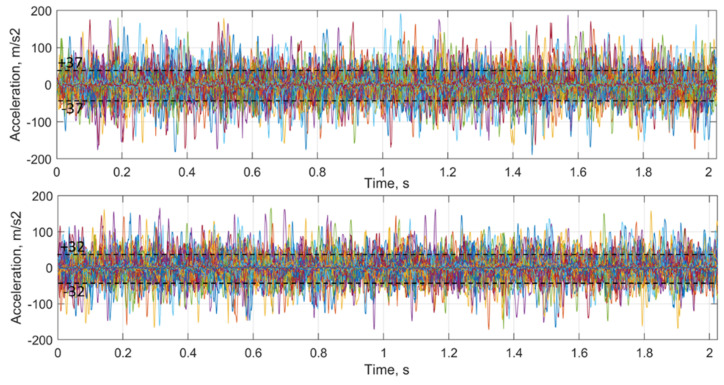
The raw time series of rail acceleration in void (**top**) and reference zone (**bottom**).

The acceleration datasets derived from deflections are presented in Figure 18 (top) for the void zone and in Figure 18 (bottom) for the reference zone. A low difference of rail accelerations for both cases with maximal values in the range of about $\pm 180\;m/s^{2}$ is notable. However, the standard deviation of the 20 most significant peaks for the void zone $\pm 37\;m/s^{2}$ is somewhat higher than for the reference one $\pm 32\;m/s^{2}$. The result is consistent with the experimental measurements of rail accelerations using acceleration sensors in the paper [4], where the maximal accelerations in the void zone were even lower than in the reference one. The lower measured acceleration range for the photogrammetrical measurement is explained with the measurement frequency of the videorecording compared to that of sensitivity range of the acceleration sensors.

In order to carry out on-board monitoring of voided sleepers, the decision rules should be obtained that enable, based on the acceleration measurement as input, to decide the severity degree of the void damage as the output. Here, we present a way to establish a decision rule. Three levels of severity are considered: void-free zone, void with depth less 3 mm, and void with more than 3 mm depth. The data are labelled in the corresponding classes: REF, HL1 and HL2. The data are randomly split in 70/30 training and test datasets and a fivefold cross-validation scheme was used.

**Figure 19:**
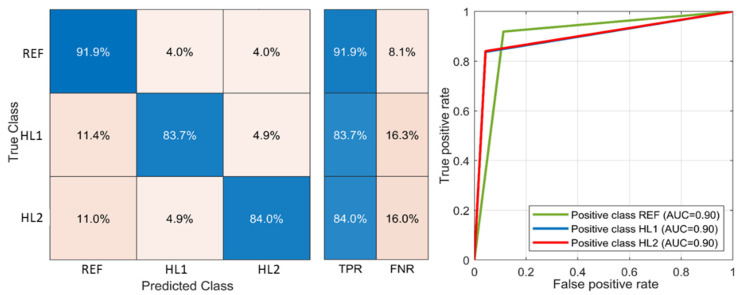
The misclassification matrix (**left**) and ROC-plot (**right**) for fine KNN classifier.

The cubic SVM and fine KNN classifiers were used to obtain the optimal decision boundaries between classes of sleeper support state. The KNN algorithm is a robust and simple classifier [100]. It is based on a non-parametric and instance-based learning algorithm that does not explicitly learn a model but exploits training instances which are subsequently used for the prediction phase. The advantage of the KNN algorithm that it is a simple model training but the weakness is its memory and computation costs during the test since it requires running down the whole dataset. Another disadvantage is the sensitivity to noisy data, outliers and missing values. Figure 19 presents the confusion matrix (left) and ROC-plot (right) for the fine KNN classifier. Each row of the matrix represents the values in a predicted class, whereas each column represents the values in the actual class. The class REF has a higher true positive rate (TPR) 91.9% than other classes. The misclassification error as the false negative rate (FNR) of the classes HL1 and HL2 is about 16%. The ROC curve (receiver operating characteristic curve) in Figure 19, left shows the performance of a classification model at all classification thresholds for the positive classes REF, HL1 and HL2. The AUC (area under the ROC curve) metric provides an aggregate measure of performance across all possible classification thresholds. The AUC for all three classes is the same 0.9; however, the ROC curve for the REF class shows better classification for high threshold values.

**Figure 20:**
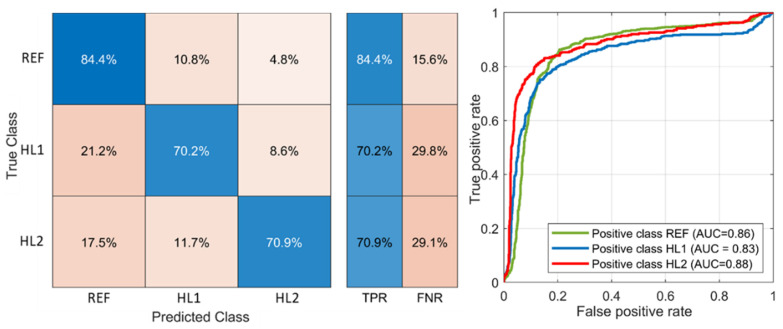
The misclassification matrix (**left**) and ROC-plot (**right**) for CUBIC SVM classifier.

The SVM classifier finds the optimal decision boundary with regard to the bias variance trade-off. It stays accurate to the training points and makes an accurate prediction with new points. The SVM presents the advantages, compared to other machine learning techniques, that it can make both linear and non-linear classifications and can better deal with outliers than KNN. The classification results for cubic SVM is presented in Figure 20. The TPR rates for HL1 and HL2 classes are noticeably lower than for the KNN classifier due to a high misclassification rate with the REF class. The ROC curves show the best classification performance for the positive classes REF and HL2.

**Figure 21:**
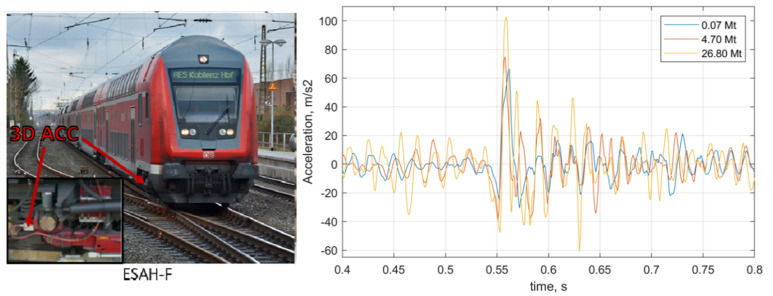
On-board ABA acceleration measurements on a common crossing [101].

To prove the applicability of the developed method of void identification, the trained statistical models were applied for a real on-board measurement. The sleeper support conditions on a common crossing were estimated using on-board inertial measurements on a regular train (Figure 20, left) [101]. Thereby, the measurements from three different moments of common crossing lifecycle were classified: 0.07 Mt, 4.7 Mt and 26.8 Mt, corresponding to the time shortly before the crossing replacement. The first class with 0.07 Mt is considered without void damage. The acceleration measurement fragments are presented in Figure 21, right. Before the classification, the time series were converted to the feature extraction network sampling rate and length, and a 200 Hz low-pass filtered to exclude the relative high impact due to crossing rails.

The classification results for the SVM and KNN classifiers are presented in Table 2. Different to the classification with track-side data (Figure 19 and Figure 20), the classification accuracy is not so optimistic. The SVM model provides the maximal accuracy 58% for the class REF. However, the classes HL1 and HL2 cannot be differentiated. The KNN model provides better classification for the class HL2 with an accuracy of 65%; however, the class HL2 is misclassified. The classification of class REF is lower than for the SVM model. Nevertheless, the classification results show prevailing REF class for 0.07 Mt measurements and HL1 with HL2 classes for the other measurements.

## 6. Discussion

The research review presents many approaches on track-side and on-board track conditions identification. However, only on-board inertial measurements could be a cost-effective and frequent method for the sleeper support condition estimation. Many studies demonstrate that the on-board measurements from regular trains can provide the comparable condition estimation as the exact but expensive measurement trains. Thereby, the main difficulty is the correct interpretation of the inertial measurements. Other studies present a successful application of artificial intelligence methods for railway engineering applications.

The mechanical modeling shows that the track dynamic behavior in the void zone was different to the equivalent geometrical irregularity specific pattern. The pattern is related to one or more dynamic impacts of the rail-sleepers grid to the ballast bed during void closure. The impacts appear in the zone before the wheel reaches it. The applied wavelet scattering to modelled on-board accelerations allowed to create the framework for the automatic extraction of significant features of the voided zone. Additionally, modelling shows that the dynamic behavior due to the voided zone is present in both track-side and on-board measurements.

The statistical data about the dynamic behavior of railway track in many voided zones were collected using high-speed video cameras. The developed computer vision software allowed to extract exact and free-from-interference multipoint information about the rail vibrations. The method is simpler and more efficient than the conventional LVDT method, which allowed quick and cost-effective information collection from many problem zones.

The collected statistical information was used to train the machine learning classifier models. The time series is separated in two independent variables: the vector of maximal sleeper void and the corresponding rail acceleration series dataset. Thereby, the Winkler-line rail deflections process is removed from rail deflections to avoid the bias to the predictor variable. An additional reason for the high-pass filtering is the intention to investigate the vibration information that is the same both for the track-side and on-board measurements. The model testing using a fully independent dataset demonstrated good enough classification results.

However, the classification with the real on-board measurements of an axle-box acceleration on a common crossing was not so optimistic. There are many explanations for the insufficient good classification results of the on-board axle-box measurements on the common crossing. The mechanical explanation is that the measurements on the common crossing have the influence of the complicated geometrical irregularities that influence the wheel trajectory. The wheel accelerations depend on the long-wave structural irregularity, short-wave wear irregularity under the influence of lateral wheel position and the wheel profile irregularities itself [52,53,96]. Therefore, the void interaction cannot be good enough differentiated with the classifiers trained only for void case. The model training for combined rail defects or irregularities as presented in [89] would probably improve the classification results.

The statistical reason of insufficient classification results is that only three measurements were used for the void estimation. Many experimental studies on wheel and rail common crossing interaction show that the axle-box acceleration measurements are subjected to high random variation due to many influencing factors. Thus, sleeper condition estimation with many different on-board measurements would bring more certainty to the classification results. The bad differentiation between the HL1 and HL2 for both classes can also be explained with unknown true state of the sleeper support condition for 4.7 Mt and 26.8 Mt since the tamping works produced are not known. The good support condition with the low void can be assumed for the measurement 0.07 Mt. However, even in this case, an insignificant void cannot be excluded due to impact interaction in the crossing zone that causes quick differential settlements.

## 7. Conclusions

The presented research shows the possibility to identify the void irregularity and classify its severity using both track-side and on-board measurements. The research is based on the data-driven approach supported by a mechanical model feature selection. The classification using simple machine learning methods shows good enough results for isolated void irregularities. However, the identification results at switch locations are less convincing due to the multiple other interactions taking place. The classification results could be further improved by using more advanced deep-learning methods. On the other hand, the application of the mechanical model for multiple irregularities could improve the data-driven classification. The following partial conclusions of the research can be proposed:

(1) One or more dynamic impacts of the rail-sleepers grid to the ballast bed occurs while void closure happens.
(2) The track dynamic behavior in the void zone is different to the geometrical irregularity-specific pattern.
(3) The similar dynamic behavior due to the voided zone is present in both track-side and on-board measurements.
(4) The developed framework for the wavelet scattering feature extraction together with mechanical modeling allow to select the significant features for the void zone.
(5) The application of machine learning methods allows good enough automatic recognition of the void zones.
(6) The pre-trained model testing for on-board measurements from a common crossing zone shows a moderate result of the void identification.

## Figures and Tables

**Table 1:** Parameters of the dynamic model.

Vehicle Parameter	Value	Track Parameter	Value
Car body mass for 1 wheel	$\;M_{B}=10,000$ kg	Rail meter mass (beam 1)	$m_{1}=$60 kg/m
Unsprung wheel mass	$M_{U}=650$ kg	Rail bending stiffness (beam 1)	$EI_{1}=6.4$ MN·m^2^
Contact stiffness	$k_{c}=2.4\times 10^{9}$ N/m	Fastenings foundation coefficient (layer1)	$k_{1}=200\;$MN/m^2^
Contact damping	$\eta_{c}=155\times 10^{3}$ N s/m	Fastenings damping (layer 1)	$\eta_{1}=1$85 kN·s/m^2^
Suspension stiffness	$k_{v}=1.1\times 10^{6}$ N/m	Sleeper meter mass (beam 2)	$m_{2}=$300 kg/m
Suspension damping	$\eta_{v}=13\times 10^{3}$ N s/m	Sleeper layer bending stiffness (beam 2)	$EI_{2}=0.01$ MN·m^2^
		Sleeper-ballast foundation coefficient (layer 2)	$k_{2}=600\;$MN/m^2^
		Sleeper-ballast damping (layer 2)	$\eta_{2}=7$5 kN·s/m^2^
		Ballast meter mass (beam 3)	$m_{3}=$500 kg/m
		Ballast-subgrade layer bending stiffness (beam 3)	$EI_{3}=2.2$ MN·m^2^
		Ballast-subgrade foundation coefficient (layer 3)	$k_{3}=200\;$MN/m^2^
		Ballast-subgrade damping (layer 3)	$\eta_{3}=680$ kN·s/m^2^

**Table 2:** The classification results for the SVM and KNN classifiers.

True Class	SVM Model Prediction, %			KNN Model Prediction, %		
	REF	HL1	HL2	REF	HL1	HL2
**0.07 Mt**	58	36	6	47	28	25
**4.7 Mt**	26	52	22	38	19	43
**26.8 Mt**	15	51	34	7	28	65

## Data Availability

The data presented in this study are available on request from the corresponding author. The data are not publicly available due to the confidentiality.
